# Adequacy of Visual Inspection with Acetic Acid (VIA) in Comparison to Pap Smear for Cervical Cancer Screening in Urban and Rural Settings of Tanzania

**DOI:** 10.3390/cancers18172811

**Published:** 2026-08-29

**Authors:** Crispin Kahesa, Daniela Gonzalez, Kandali Samwel, Duan Loy, Cameron Klein, Abigail Shotwell, Martha Ricaurte, Julius Mwaiselage, Brenda B. Kweyamba, Asafu Munema, Monica K. Angeletti, Kessy Godwin, Elayna Brown, Gleb Haynatzki, Veenu Minhas, John T. West, Charles Wood, Peter C. Angeletti

**Affiliations:** 1 Ocean Road Cancer Institute, Dar es Salaam P.O. Box 3592, Tanzania; crispinkahesa@yahoo.co.uk (C.K.); jmwaiselage@yahoo.com (J.M.);; 2 Nebraska Center for Virology, School of Biological Sciences, University of Nebraska-Lincoln, Lincoln, NE 68583, USA; 3 Department of Biostatistics, College of Public Health, University of Nebraska Medical Center, Omaha, NE 68198, USA; 4 AbbVie,1 N Waukegan Rd., North Chicago, IL 60064, USA; 5 Department of Interdisciplinary Oncology, Louisiana State University Health Sciences Center—New Orleans, New Orleans, LA 70112, USAcwoo12@lsuhsc.edu (C.W.)

**Keywords:** human papillomavirus (HPV), human Immunodeficiency 1 (HIV-1), visual inspection with acetic acid (VIA), Pap smear, neoplasms

## Abstract

**Background/Objectives:** Tanzania is a low-to-middle-income-country (LMIC) which endures significant adverse impact from HIV-1- and HPV-associated malignancies. We investigated the performance of visual inspection with acetic acid (VIA) compared to the Pap smear. **Methods**: Screening performance was assessed in a cross-sectional study design across rural catchment clinics in Bagamoyo and Chalinze, and an urban site, Ocean Road Cancer Institute (ORCI), in Dar es Salaam. Pap smears were performed and were read in triplicate by readers blinded to HIV status and patient demographic data. Blood samples were collected for HIV status confirmation. All cytopathology, VIA, and HIV status data were compared with patient demographic factors. **Results:** Here we present results from 672 patients. Analysis of participants across the cohort revealed a high rate of HIV (25%) and exchange of “sex for money” in the rural site of Chalinze. Bagamoyo and ORCI had slightly lower HIV rates of about 16% and 12%, respectively. Despite the high HIV rates in Chalinze (25%), there was a contrastingly low-level of detectable lesions by VIA (9%) in this region. **Conclusions**: Overall, VIA-positive cases were 26% HSIL-positive, whereas VIA-negative cases were 14% HSIL-positive, suggesting that performance of VIA is limited.

## Simple Summary

Cervical cancer, caused by Human Papillomavirus (HPV), is the leading cause of death of women in sub-Saharan Africa. Progression of cervical cancer is strongly influenced by HIV coinfection. In Tanzania, cervical cancer screening is primarily achieved by visual-inspection-with-acetic-acid (VIA) rather than the Pap smear. In the present study, we investigated the adequacy of cervical cancer screening by VIA compared to the Pap smear. To perform this study, we recruited 672 women from two rural clinics in Bagamoyo and Chalinze and one urban clinic at the Ocean Road Cancer Institute (ORCI) in Dar es Salaam. Participants were given a demographic survey, blood was drawn for HIV testing, and Pap smears were taken and reviewed by readers blinded to patient data. We found that VIA was less efficient than the Pap smear. While VIA correctly identified 26% of high-grade dysplasias,14% of VIA-negative cases had high-grade abnormalities, indicating a high rate of false negatives. These results show the practical weaknesses of the VIA method.

## 1. Introduction

Human papillomavirus (HPV) is the most common sexually transmitted infection worldwide with over 200 distinct high-risk (HR) and low-risk (LR) genotypes. There are at least 12 HR-HPV types known to cause cervical and other cancers of mucosal tissues [1]. HPV remains a significant global health concern and causes at least 4.8% of all cancers, despite vaccine efforts [2,3,4,5,6,7,8,9]. In low-to-middle-income countries (LMICs), such as those in sub-Saharan Africa, HPV-associated cancers occur at a high rate [2]. Sub-Saharan Africa has only 9% of the world’s female population, but 18% of the world’s deaths due to cervical cancer [10].

HIV is strongly associated with the increased cervical cancer incidence and mortality observed in LMICs [11,12,13,14]. HIV prevalence in sub-Saharan Africa ranges from 5 to 30% [15] and HIV-associated malignancies remain a major cause of morbidity and mortality among infected patients. The combination of endemic HIV and the variable geographical distribution of HR-HPVs across sub-Saharan countries makes cervical cancer a significant challenge. Tanzania has an age-standardized cervical cancer incidence rate of 64.8 cases per 100,000 women, and an age-standardized mortality rate of 42.2 per 100,000 women due to cervical cancer [8]. Cervical cancer screening, clinical interventions, and HPV vaccination strategies remain limited in Tanzania and other sub-Saharan countries [16,17,18].

Because of the greater risk for progression of cervical neoplasms among HIV-positive women, there is significant need for effective and low-cost cervical cancer screening strategies in sub-Saharan Africa [19]. Currently, the most common approach for cervical screening in Tanzania has been visual inspection with acetic acid (VIA) rather than the Pap smear [20], primarily due to the resource and expertise costs. Thus, the present study investigates the efficacy of VIA compared to Pap smear screening of Tanzanian women, an at-risk population. We compared cervical cancer screening methods in a cross-sectional study of 672 women across rural catchment clinics in Bagamoyo and Chalinze, and an urban site: Ocean Road Cancer Institute (ORCI) in Dar es Salaam. There is significant variation in HIV infection rates and access to cervical cancer screening services across the rural and urban areas of Tanzania. In the present study, we investigated the adequacy of the VIA cervical cancer screening method among Tanzanian women from urban and rural settings in the context of high HIV infection rates [16,20,21].

## 2. Materials and Methods

### 2.1. Enrollment and Data Collection

A cross-sectional study of women from urban and rural areas was conducted between March 2015 and January 2022. The study protocol was reviewed and approved by the Institutional Review Boards of Ocean Road Cancer Institute (ORCI), Dar es Salaam, Tanzania and the University of Nebraska-Lincoln (IRB Approval #20141014709FB), following the Declaration of Helsinki ethical standards for human subject studies.

This study was conducted at the three study sites in order to represent women across a wide range of socioeconomic, educational, residential, and lifestyle backgrounds indicative of the whole country. (A) The Ocean Road Cancer Institute (ORCI) in Dar es Salaam representing urban Tanzania: Dar es Salaam is the capital of the country and has a population of about 5.4 million. ORCI is a cancer referral center that serves more than 4500 new cancer patients/year. These patients are from all over the country, and approximately 80% are in advanced stages of disease. ORCI also attends to over 15,000 follow-up cancer cases annually. The institute provides inpatient and outpatient services, including a 258-bed capacity hospital, cancer prevention services, general laboratory services, diagnostic imaging services, and radiotherapy, as well as chemotherapy infusion. (B) The Bagamoyo district hospital representing a semi-rural area: This hospital is located about 67 km north of Dar es Salaam and serves a mixed rural/urban community of >300,000 inhabitants and has 45 beds for general care. The hospital has an adjacent research laboratory, the Bagamoyo Research and Training Unit, which supports on-site clinical studies, as well as cervical cancer screening services for women from different neighboring districts. (C) The Chalinze Health Center in a rural area: This clinic is located 113 km west of Dar es Salaam and serves a community of about 35,000 people. It provides primary health care services and provides screening services for cervical cancer. All of these clinic sites provide support for people living with HIV (PLWH).

Women aged 18 and older were recruited from the three sites, and informed consent was obtained prior to sample collection. Women who were pregnant, had prior hysterectomy, or diagnosed lymphoma were excluded from the study. All participants were interviewed by trained female nurses using a structured questionnaire to collect information on socioeconomic factors, lifestyle, and reproductive history. The women had a gynecological examination, including visual inspection with acetic acid (VIA) and a conventional Pap smear. In addition, a blood sample, obtained from each woman for HIV testing, and remaining blood was fractionated into plasma and peripheral blood mononuclear cells (PBMCs).

### 2.2. Cervical VIA

VIA was performed according to WHO/IARC protocol as a routine standard of care. Acetic acid (5%) was applied to the cervix using a cotton swab and the cervix was visualized after a minute with the aid of a halogen light source [16]. The test was classified as positive if acetowhite epithelium appeared near the squamous columnar junction. The test was classified as suspicious for cancer if a cauliflower-like fungating mass or ulcer was observed on the cervix. If the cervical lining was smooth, uniform, and with no acetowhite lesion, the test was classified as negative [16].

### 2.3. Pap Smear

Pap smear collection was performed using the concave end of a standard Ayer’s spatula, and samples were evenly spread on a glass slide and sprayed with a solution containing Boonfix (ethanol, PEG and acetic acid). After fixation, slides were air-dried and stored in plastic slide containers. Slides were stained with OG-6 to stain keratinized cells, and with EA-50 for metabolic activity, followed by clearing and mounting. Three cytologists independently determined Pap smear results according to the 2001 Bethesda classification system as follows: NILM (Negative for Intraepithelial Lesion or Malignancy), ASCUS (Atypical Squamous Cells of Undetermined Significance), LSIL (Low-grade Squamous Intraepithelial Lesion), ASC-H (Atypical Squamous Cells-cannot exclude HSIL), and HSIL (High-grade Squamous Intraepithelial Lesion) [16,22]. Results that differed significantly between readers were reconciled by a second analysis. Pap smears were performed as a comparison to VIA but were not routinely provided as a standard of care in study clinics.

### 2.4. HIV Status

Medical records were the primary means to determine HIV status of study participants (under routine standard of care), but if unknown to the patient, HIV status was determined by using the Sure Check HIV rapid serologic assay, using the protocol provided by the manufacturer (ChemBio, Medford, NY, USA).

### 2.5. Statistical Analysis

All statistical analyses were carried out using SAS software, version 9.4. Risk factors for VIA-positive results (alcohol, six or more pregnancies, sex at seventeen years old or younger, birth control, sexually transmitted infection, and HSIL) were separately calculated for the total population, among HIV-positive, and among HIV-negative women. Univariate logistic regression was used to initially identify potential risk factors associated with VIA-positive results. Factors that were statistically significant in the univariate analyses or were thought to be important predictors based on previous knowledge were included in multivariate analyses, estimating the odds ratio (OR) with 95% confidence interval (CI). Performance of VIA versus HSIL was analyzed with Fischer’s Exact test.

## 3. Results

**Figure 1:**
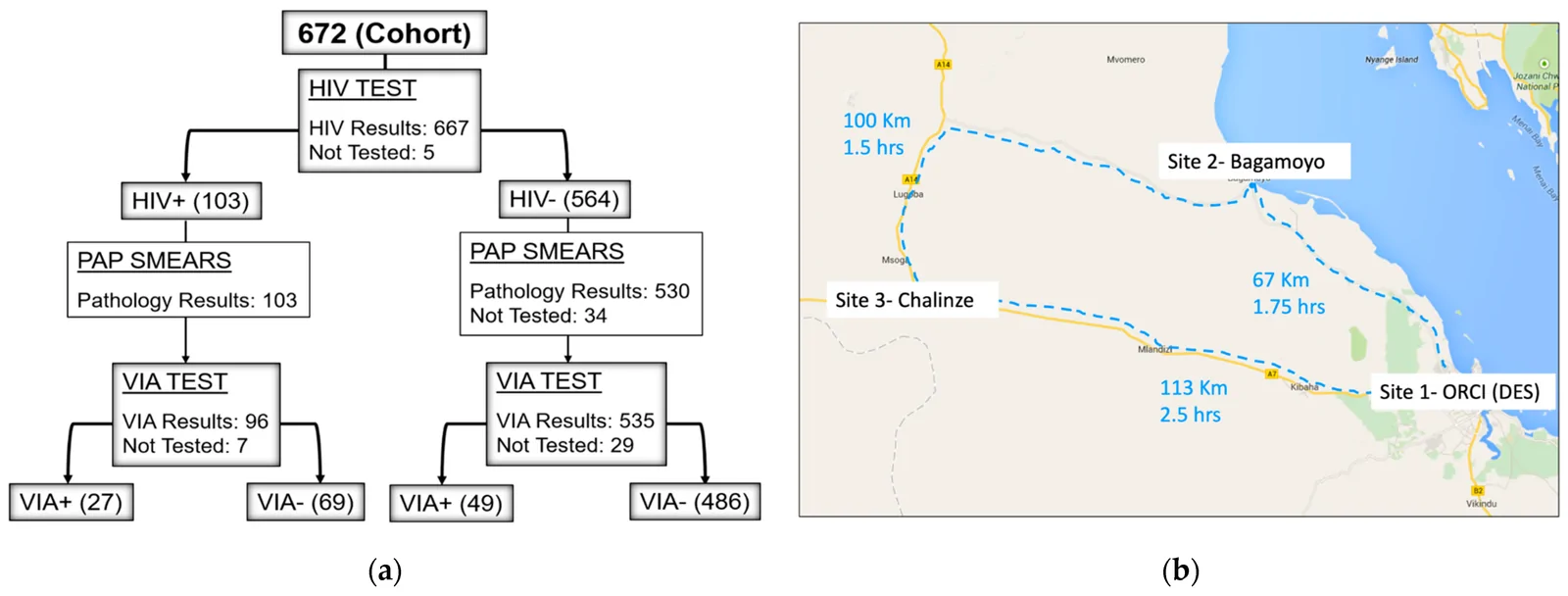
(**a**) Flowchart summarizing the cervical cancer screening implemented for all 672 women in the three Tanzanian study sites. Study participants were tested for HIV status, followed by Pap smear and then VIA analysis. Data loss due to inadequate tissue sampling or screening opt-outs are indicated at each stage. (**b**) Map of women’s clinic catchment sites at ORCI, Bagamoyo, and Chalinze.

In this study, a total of 672 Tanzanian women were recruited from three different locations: the Bagamoyo (170 women) and Chalinze (127 women) rural sites, and an urban site, ORCI (375 women), in Dar es Salaam. The recruitment and screening strategy implemented across in all participants is summarized in a flowchart (Figure 1a). This consisted of a gynecological examination, including VIA and a conventional Pap smear, as well as a blood sample for HIV testing and viral loads. Of the 667 patients tested, 103 were HIV-positive, and 564 were HIV-negative. Both Pap smear and VIA were performed for a total of 631 patients. Locations and distances between patient catchment sites, ORCI, Bagamoyo, and Chalinze, are mapped in Figure 1b. Sociodemographic factors, reproductive/sexual history, and medical conditions of women attending the cervical cancer screening clinics at each of the study sites are summarized in Table 1. HIV-positive rates were 15% overall, 12% at ORCI, 16% at Bagamoyo, and 25% at Chalinze catchment sites, respectively. The consumption of alcohol was low across all participants (11–19%). Birth control usage rates were high (64–78%) across all three study sites. Chalinze had a 9% VIA-positive rate, whereas ORCI and Bagamoyo were 12% and 14% respectively. Yet, Pap smears revealed Chalinze had the highest rates of HSIL (18%), whereas ORCI and Bagamoyo were 11% and 10% HSIL respectively.

In Table 2, the risk factors for VIA-positive results among women in Tanzania are summarized. Overall, women in the 25–34 age group had 2.3 times (95% CI, 1.1–4.8) higher odds of being VIA-positive when compared with 45-years-or-older women. The risk increased with decreased household income; women with low income had 1.2 times higher odds (95% CI, 0.4–4.0) of being VIA-positive compared with those reporting having high household income. Education level and birth control were not associated with a risk of VIA positivity. Age at sexual debut represented a risk factor; women who had first intercourse at the 18–20 years old group had 2.5-times-higher odds (95% CI, 1.0–5.7) of being VIA-positive in comparison to women 21 years old or older. Overall, being HIV-positive was the strongest risk factor for a VIA-positive result (OR, 4.1; 95% CI, 2.3–7.5). In addition, the risk increased with increasing abnormality in Pap smear results, as would be expected. Women diagnosed with HSIL (95% CI, 1.3–9.9) and LSIL (95% CI, 1.0–6.2) had 3.6- and 2.5-times-higher odds of being VIA-positive, respectively. When considering only HIV-positive women, the odds ratios increased to 4.9 (HSIL) and 3.3 (LSIL), respectively.

Our comparison of VIA versus Pap smear revealed that VIA performance varies significantly based on HIV status (Table 3). The VIA test showed that HIV-positive women were more likely to be HSIL+ (*p* = 0.0065, OR = 5.28) than HIV-negative women. Among VIA-negative cases, detection of HSIL+ showed that false negative detection was prominent and that HIV status did not significantly affect that detection (*p* = 0.8380, OR = 0.85). Significant differences in VIA performance as a function of HIV-positive status were seen among the VIA+ groups in the overall cohort, as well as in ORCI and Bagamoyo, but in Chalinze, VIA detection failed to show significance under any HIV status. These results suggest that the VIA method is susceptible to false negatives. In our study, there may have been much less effective use of the VIA method at the Chalinze site. Overall, these results suggest limitations of VIA as a standalone screen as compared to Pap smear.

**Figure 2:**
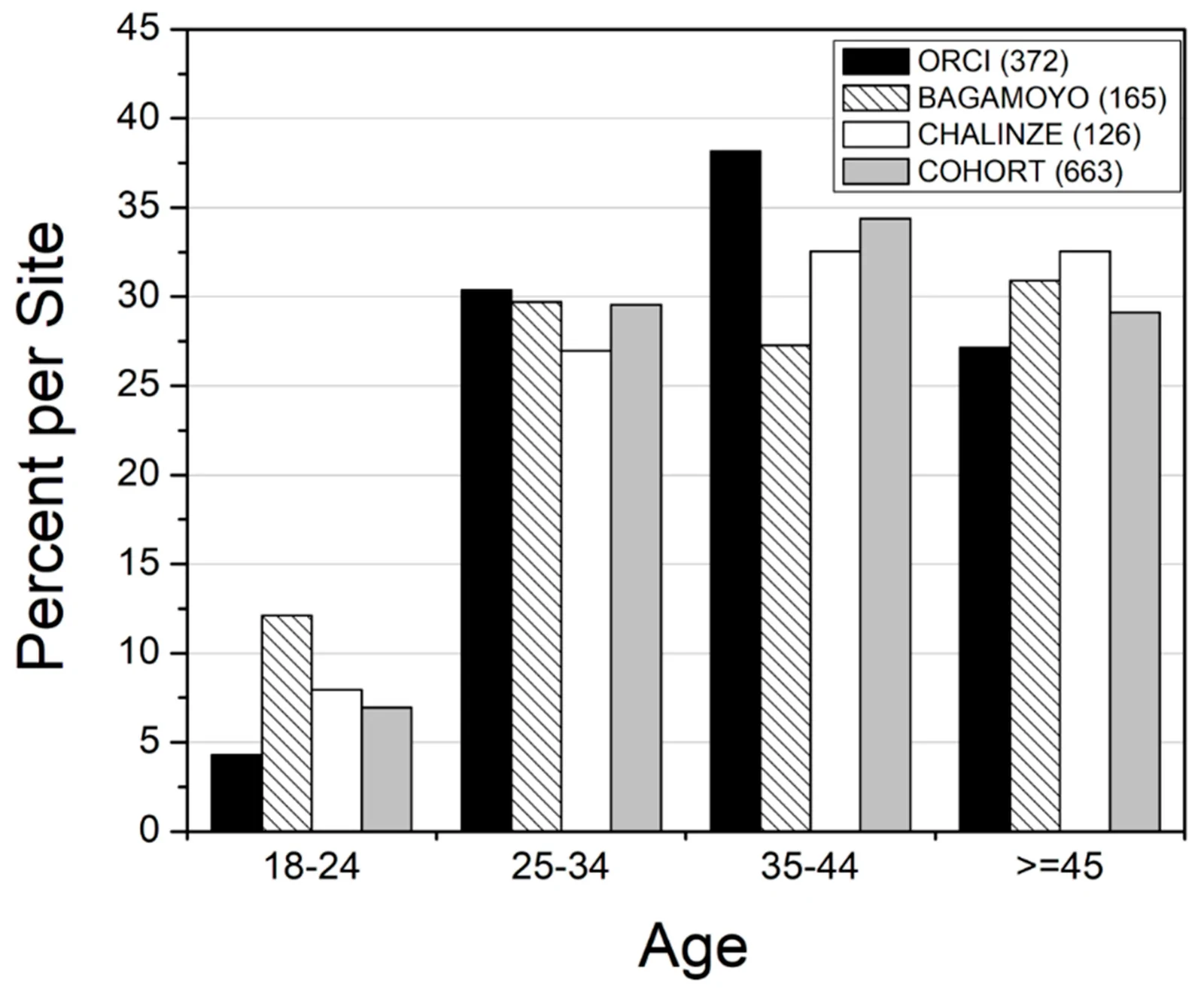
The age distribution of participants per study sites. Most of the women recruited to the study were between 25 and 44 years old.

**Figure 3:**
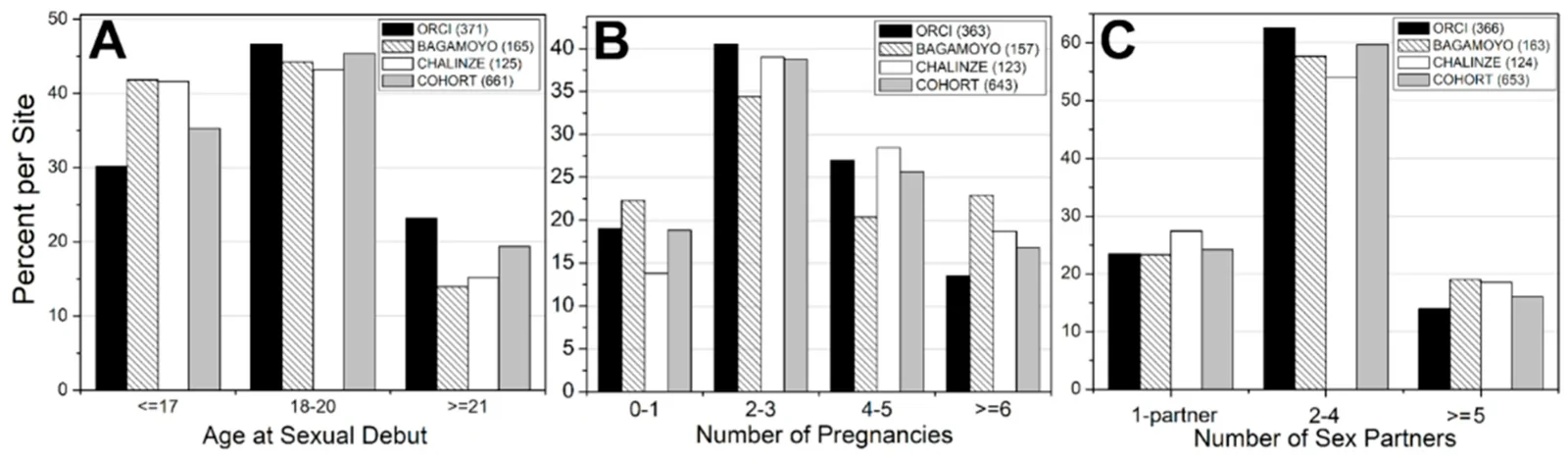
Sexual and reproductive characteristics of the study population across cohort and catchment sites: (**A**) age at sexual debut, (**B**) number of pregnancies, and (**C**) number of sex partners. The percent per site is indicated on the left axis and frequencies per site are indicated in the upper right of each graph.

The age distribution across each of the catchment sites was relatively similar, as shown in Figure 2. We found that 64% of women were in the 24–44-year-old age group, 7% were in the 18–24-year-old group, and 29% were in the 45-years-or-older group age. Figure 3 summarizes age of sexual debut, number of pregnancies, and number of sex partners across the catchment sites. We observed that the percentage of women who have their sexual debut at 17 years or younger was higher in the rural areas of Bagamoyo and Chalinze than in the urban area (ORCI), at 42% versus 30%, respectively (Figure 3A). The number of pregnancies equal to or greater than six was higher in the rural areas (Bagamoyo and Chalinze) compared to the urban area (ORCI) (Figure 3B). Regarding the number of sexual partners, the percentages were relatively similar for 1, 2–4 and ≥5 partners across the three study sites (Figure 3C).

**Figure 4:**
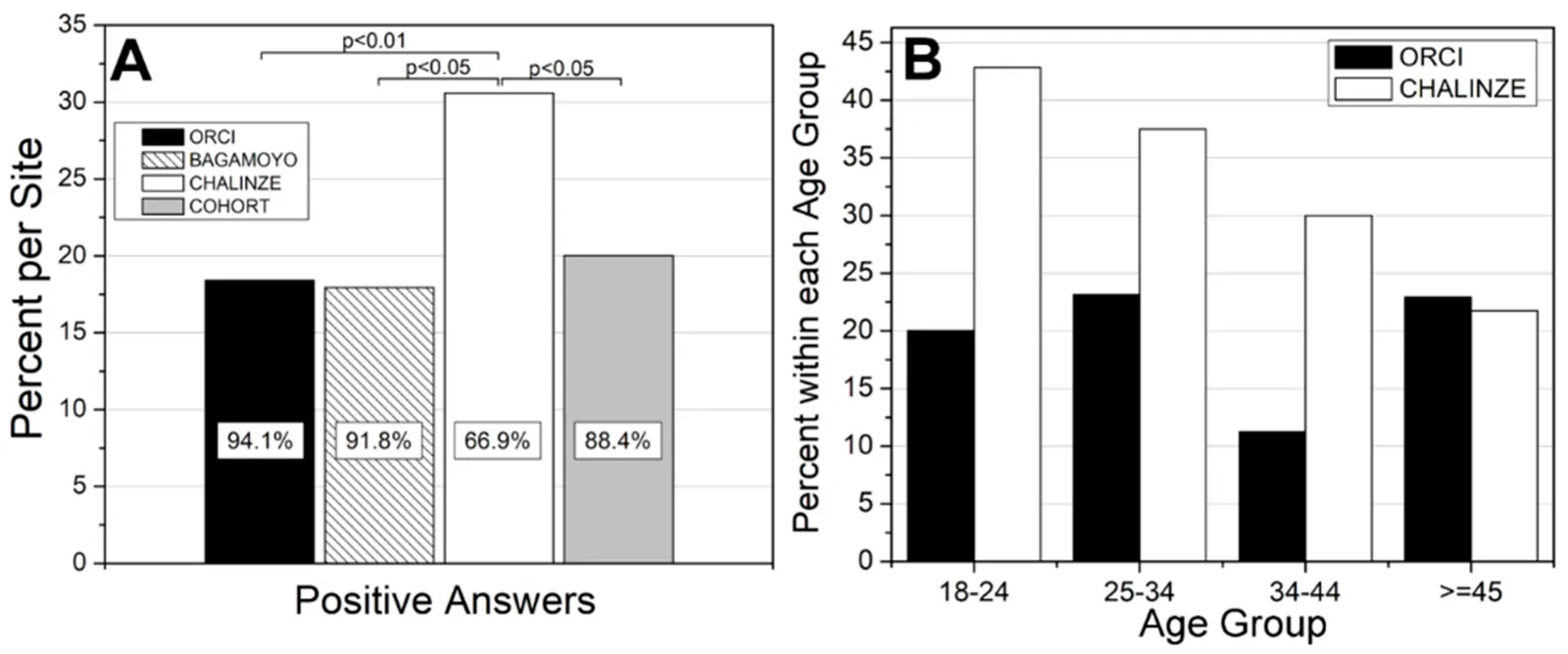
The percent per site positive answers to the query: (**A**) ‘Sex for money’. The numbers inside each bar correspond to the percent respondents at each site. There were significant differences between Chalinze when compared with the other sites ( *p* < 0.05 and *p* < 0.01). (**B**) Sex for money per study site and age groups. The percentages represent ‘sex for money’ positive answers per site within each age group.

The percentages of positive responses to the question of ‘sex for money or gifts’ is shown in Figure 4. We found that 30% of women in Chalinze responded positively to this question, which was significantly higher than respondents in the urban site (ORCI, 18%), (*p* < 0.01). We also found that participants from Chalinze had the lowest response rate to the ‘sex for money or gifts’ question (67%), which indicates that the 30% positive responses rate is likely to be an underestimation (Figure 4A). Furthermore, when answers were grouped by age, the comparison between ORCI and Chalinze showed that ‘sex for money’ positive answers were higher among younger women (18–24-year-olds) (43%) and decreased among those 45 years old or older to 22%, (Figure 4B).

**Figure 5:**
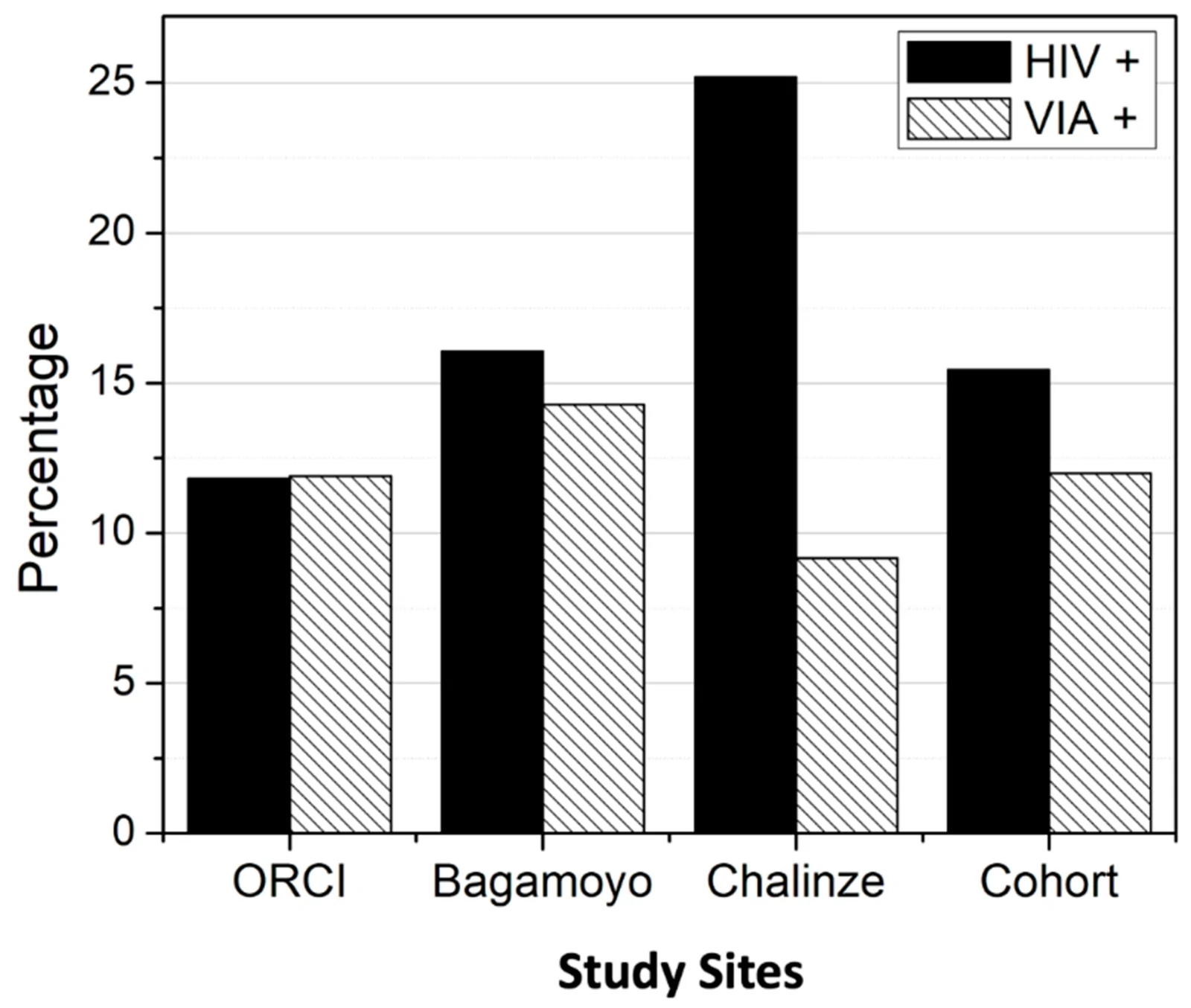
HIV- and VIA-positive percentages per study sites. Chalinze presented the highest HIV-positive rate (25%), while ORCI and Bagamoyo presented lower values of 12% and 16%, respectively.

We analyzed the HIV and VIA positivity rates per study site and for the total cohort (Figure 5). The results revealed a very high rate of HIV positivity (25%) in the rural site of Chalinze, which coincides with the high rate of ‘sex for money or gifts’ among 18–24-year-old women. Bagamoyo and ORCI had lower HIV rates of about 16% and 12%, respectively. Despite the high HIV rates in Chalinze, there was a relatively low level of detectable lesions (9%) by VIA in this region, while ORCI and Bagamoyo presented VIA-positive results of 12% and 14%, respectively.

**Figure 6:**
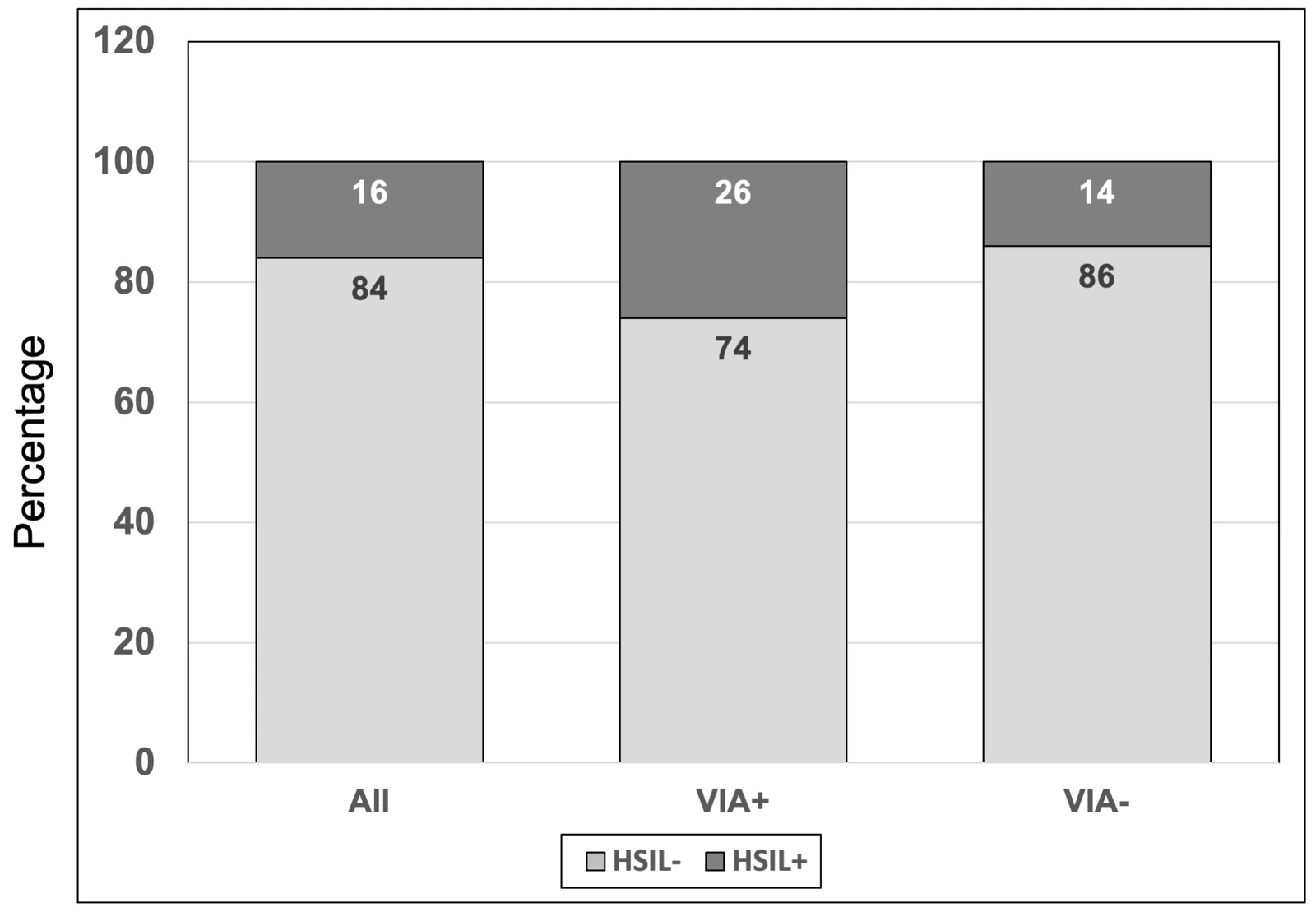
HSIL status was compared to all (n = 421), VIA+, or VIA− status in the total cohort. The percentage of HSIL+ is indicated by the dark gray bar and HSIL− is indicated by the light gray bar. Among VIA+ women, 26% of cases were HSIL+, and 74% were HSIL−, whereas 14% of HSIL+ was found among VIA− cases and 86% of HSIL− was found among VIA− cases.

We compared the percentage of abnormal cytology (HSIL to LSIL) among VIA-positive and negative patients (Figure 6). The results showed that among VIA-negative women, 14% of HSIL cases were missed by the VIA method. In contrast, HSIL was observed in 24% of VIA-positive cases. The relatively high number of HSIL cases among VIA-negative patients suggests potential false negatives by the VIA method.

**Figure 7:**
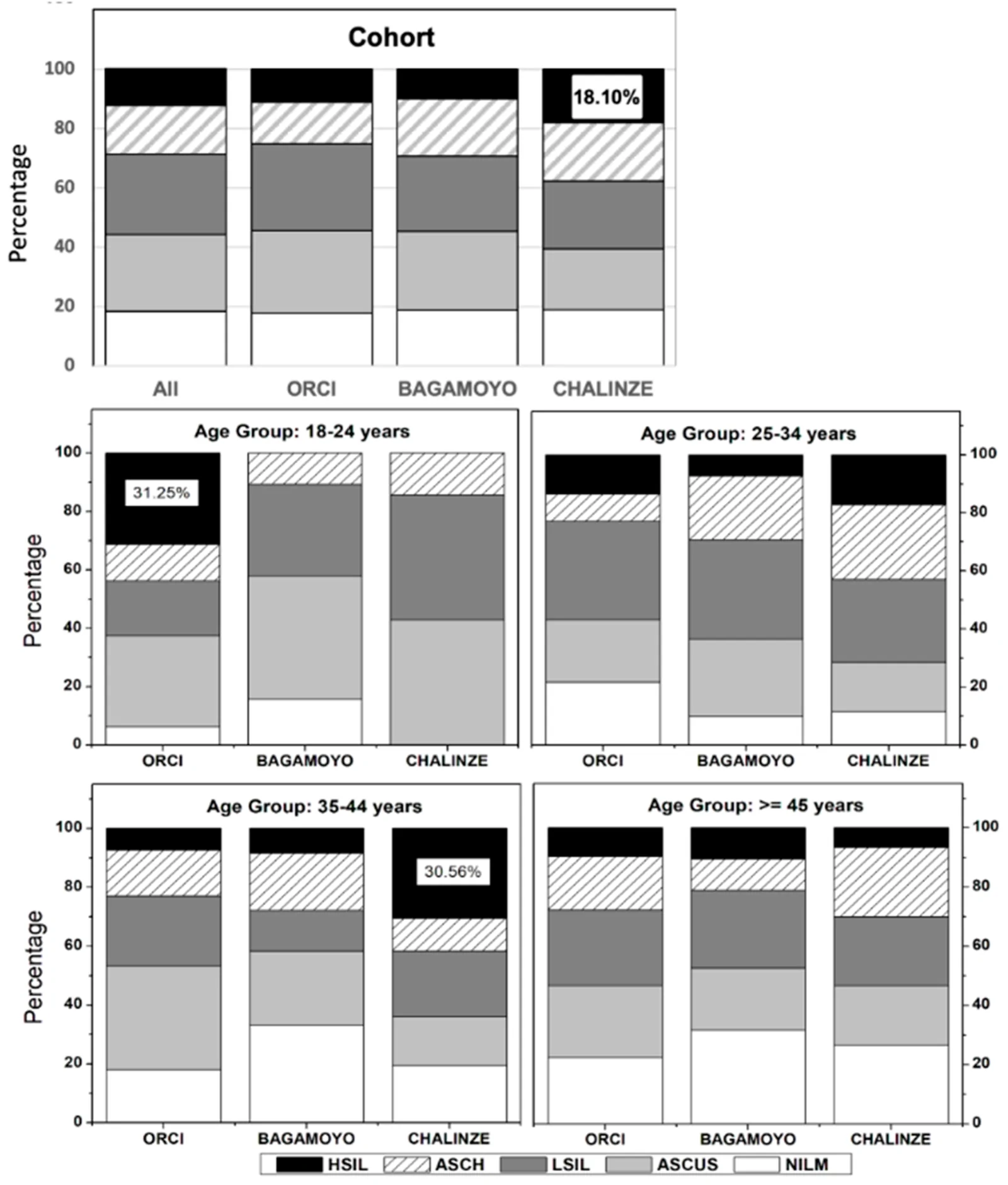
The distribution of cytopathology among VIA-negative women across age group, and collection sites in Tanzania. The top panel displays the cytopathology distribution for the total cohort across study sites, whereas the lower panels display each age group distribution. The percentage of cytology is indicated below the graphs; NILM, ASCUS, LSIL, ASCH, or HSIL.

The distribution of cervical cytology across age groups and catchment sites is shown in Figure 7. Cytology of VIA-negative cases were graphed as a percentage of NILM, ASCUS, LSIL, ASC-H, or HSIL in each of the age groups and across each of the catchment sites, ORCI, Bagamoyo and Chalinze, and the total cohort. Notably, HSIL-positive cases were absent in the 18–24-year-old age group in Bagamoyo and Chalinze, yet HSIL positives were found more commonly in older age groups. The percentage of HSIL was high in Chalinze (30%) among 35–44-year-olds and among 18–24-year-olds at ORCI. In the cohort overall, LSIL cases were 27% and HSIL cases were 12%. Though HSIL cases were 18% in Chalinze (Figure 7), VIA detection was only 8% in Chalinze (Figure 5), which underscores the problem with VIA reliability.

**Figure 8:**
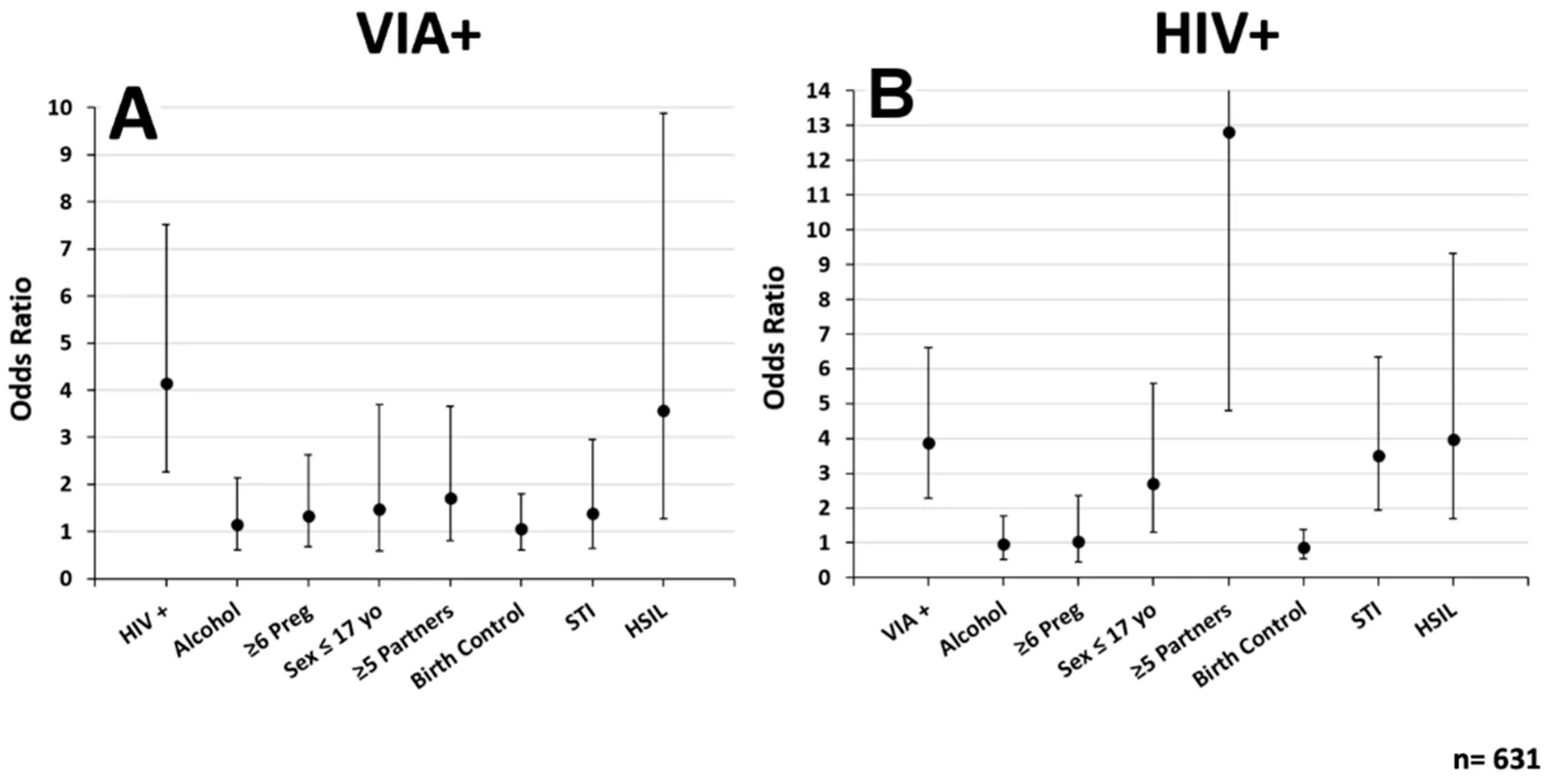
The odds ratios and risk factors across the cohort was graphed for: (**A**) VIA-positive women with respect to HIV positivity, alcohol, greater than 6 pregnancies, sex at 17 years old or younger, greater than equal to 5 partners, birth control, sexually transmitted infections, or HSIL, and (**B**) HIV-positive women with respect to VIA positivity, alcohol, greater than 6 pregnancies, sex at 17 years old or younger, greater than or equal to 5 partners, birth control, sexually transmitted infections, or HSIL.

Figure 8 shows the risk factors for women in Tanzania who are VIA-positive (Figure 8A) and HIV-positive (Figure 8B). HIV-positive status (OR, 95% CI: 4.14, 2.27–7.53) as well as HSIL pathology (OR, 95% CI: 3.56, 1.28–9.89) are highly associated with VIA positivity. The risk factors for HIV positivity are also highly related to VIA positivity (OR, 95% CI: 3.88, 2.28–6.61), having sex at age 17 years old or younger (OR, 95% CI: 2.70, 1.30–5.58), having five or more sexual partners (OR, 95% CI: 12.82, 4.79–34.30), presenting with a sexually transmitted infection (STI), such as HPV, herpesvirus, or chlamydia (OR, 95% CI: 3.51, 1.94–6.34), and presenting with HSIL pathology (OR, 95% CI: 3.98, 1.70–9.33). Overall, our results show that VIA screening misses at least 14% of HSIL+, which could be a clinically important weakness.

## 4. Discussion

In this study, we found a significantly higher HIV-positive rate (25%) among women at the rural site of Chalinze (Table 1, Figure 5), compared to the ORCI (12%) or Bagamoyo (16%) study sites. Chalinze also had very high rates of HSIL (18%) compared to ORCI (11%) and Bagamoyo (10%), which is consistent with the higher HIV rates in Chalinze (Table 2). The estimated prevalence of HIV in Tanzania and neighboring countries varies significantly by province (5–15%) [23]. The high HIV incidence we observed was expected since our recruitment occurred at clinic sites that supported routine care for PLWH. Our observation of high levels cytological abnormalities among this cohort is not surprising in light of the fact that HIV is one of the strongest drivers of HPV-associated disease [11,12,13,14].

We found a relatively high rates of positive responses to the question of ‘sex for money or gifts’ (Figure 4A). Chalinze had the highest rate of positive answers (30%), compared to ORCI (18%) (*p* < 0.01). Consistent with these results, other studies find the prevalence of transactional sex in sub-Saharan Africa ranges from 6 to 28%, depending on region [24]. We also found that 43% of younger women (18–24-year-old) in Chalinze engaged in transactional sex, whereas only 20% of ORCI respondents of the same age reported transactional sex (Figure 4B). The high rates of HIV and transactional sex in Chalinze were most likely due to poverty and that the town lies on a major trucking route close to the Zambian border.

Our analysis of HIV and VIA positivity rates per study site revealed that while HIV rates were 25% among Chalinze participants, the VIA-positive rate was only 9% (Figure 5). Yet, Pap smear analysis revealed Chalinze had high levels of HSIL (18%) among young women screened in Chalinze (Table 1). This result clearly demonstrates the limitations of the VIA in an at-risk population. It is possible that reason for the low VIA performance at the rural Chalinze site had to do with lack of proper training of staff to perform VIA at that site.

Within our study population, VIA screening fell short of reliable detection, compared to the Pap smear. The rationale for adoption of VIA over Pap smears in LMICs is understandably based on; cost, infrastructure, available expertise, clinical lab turnaround time, and the need for immediate treatment decisions. Yet, our findings showed that about 14% of HSIL was detected among VIA-, suggesting that the VIA is susceptible to false negatives (Figure 6). In contrast, Pap smear analyses provided a comprehensive view of cytopathology (Figure 7), which is superior for clinical decision making. The biggest concern for the VIA approach was that its detection fell short in Chalinze, where the HIV-positive rate was 25%, and HSIL was 30% among 35–44-year-old women (Figure 5 and Figure 7). This is exactly the high-risk population which needed the benefit of effective cervical cancer screening most. The high rates of cytological abnormalities in this cohort are consistent with the relatively high HIV positivity (15%).

Multiple risk factors for VIA-positive women were evaluated in this study, including HIV-positive status and HSIL diagnosis (Figure 8A). The risk factors associated with HIV positivity included: VIA positivity, having sex at age 17 years old or younger, having five or more lifetime sexual partners, presenting with an STI, and presenting with HSIL cytology (Figure 8B). HIV-positive status is the strongest factor associated with cervical pathology [11,12,14,25] observed in our study cohort and emphasizes the need for enhanced cervical cancer screening methods for women living with HIV.

## 5. Conclusions

Our analysis of VIA versus Pap smear revealed that the VIA performance was inconsistent. For example, we found that VIA screening was better at detection of HSIL+ among HIV+ women (Table 3). However, the assay suffers from both false positives and falls negatives. Given these weaknesses, combining VIA with an objective molecular triage methods, such as HPV PCR testing, could reduce overtreatment while preserving higher sensitivity in HIV-positive populations.

From our observations, the limitation with VIA were not only due to differential HIV status; it is also likely to be related to the fact that acetic-acid whitened cellular abnormalities are microscopic in size. Other studies have reported similar shortcomings for VIA, primarily due to unreliable efficiency [26,27,28,29,30,31,32]. We also concluded that it is possible that the low VIA performance, particularly in Chalinze, could have been related to inconsistencies in training VIA screeners. There is clearly a need for innovative and cost-effective approaches that can be readily implemented in LMICs. New guidelines from the American Cancer Society and the WHO similarly recommend HR-HPV DNA testing every 5 years as the preferred screening method for 30–65-year-olds, with self-sampling as an option [33,34]. The Pap smear is still recommended for 20–29-year-old women every 3 years. Adapting this advice to LMICs would be challenging due to the cost of HR-HPV tests. Furthermore, over-reliance on HPV DNA testing alone may lead to overtreatment [31]. Alternatively, a combination of simplified HPV DNA testing with basic cytology may be the least expensive and most effective solution for LMICs [16], but workable solutions still need further development.

## Figures and Tables

**Table 1:** General characteristics of study participants, overall and per study site.

	ALL WOMEN (n = 672)		ORCI (n = 375)		BAGAMOYO (n = 170)		CHALINZE (n = 127)	
	n	%	n	%	n	%	n	%
**Sociodemographic factors**								
Age								
18–24	46	6.94	16	4.30	20	12.12	10	7.94
25–34	196	29.56	113	30.38	49	29.70	34	26.98
35–44	228	34.39	142	38.17	45	27.27	41	32.54
≥45	193	29.11	101	27.15	51	30.91	41	32.54
Place of residence								
ORCI	375	55.80	-	-	-	-	-	-
Bagamoyo	170	25.30	-	-	-	-	-	-
Chalinze	127	18.90	-	-	-	-	-	-
Education level								
No formal education	69	10.36	25	6.70	26	15.66	18	14.17
Primary	381	57.21	198	53.08	102	61.45	81	63.78
Secondary	155	23.27	105	28.15	28	16.87	22	17.32
College/University	61	9.16	45	12.06	10	6.02	6	4.72
Household income								
Low	213	32.97	104	28.34	67	43.23	42	33.87
Medium	202	31.27	137	37.33	25	16.13	40	32.26
Variable	176	27.24	86	23.43	56	36.13	34	27.42
High	55	8.51	40	10.90	7	4.52	8	6.45
Religion								
Islam	345	52.11	165	44.72	110	66.27	70	55.12
Christianity	317	47.89	204	55.28	56	33.73	57	44.88
Alcohol								
Yes	106	16.13	71	19.24	19	11.52	16	13.01
Never	481	73.21	254	68.83	128	77.58	99	80.49
Not anymore	70	10.65	44	11.92	18	10.91	8	6.50
**Reproductive and sexual history**								
Pregnancy number								
≥6	108	16.80	49	13.50	36	22.93	23	18.70
4–5	165	25.66	98	27.00	32	20.38	35	28.46
2–3	249	38.72	147	40.50	54	34.39	48	39.02
0–1	121	18.82	69	19.01	35	22.29	17	13.82
First sex at age								
≤17	233	35.25	112	30.19	69	41.82	52	41.60
18–20	300	45.39	173	46.63	73	44.24	54	43.20
≥21	128	19.36	86	23.18	23	13.94	19	15.20
Sexual partner number								
≥5	105	16.08	51	13.93	31	19.02	23	18.55
2–4	390	59.72	229	62.57	94	57.67	67	54.03
1	158	24.20	86	23.50	38	23.31	34	27.42
Sex for money or gifts								
Yes	119	20.03	65	18.41	28	17.95	26	30.59
No	475	79.97	288	81.59	128	82.05	59	69.41
Birth control								
Yes	463	71.23	259	70.38	127	78.40	77	64.17
No	187	28.77	109	29.62	35	21.60	43	35.83
**Medical conditions**								
Diagnosed with STI								
Yes	60	9.62	32	8.99	13	8.55	15	12.93
No	564	90.38	324	91.01	139	91.45	101	87.07
HIV status								
HIV-positive	103	15.44	44	11.83	27	16.07	32	25.20
HIV-negative	564	84.56	328	88.17	141	83.93	95	74.80
VIA status								
VIA-positive	76	11.99	42	11.90	23	14.29	11	9.17
VIA-negative	558	88.01	311	88.10	138	85.71	109	90.83
Pap smear results								
HSIL	81	12.16	41	11.11	17	10.00	23	18.11
ASC-H	110	16.52	52	14.09	33	19.41	25	19.69
LSIL	180	27.03	108	29.27	43	25.29	29	22.83
ASCUS	173	25.98	102	27.64	45	26.47	26	20.47
NILM	122	18.32	66	17.89	32	18.82	24	18.90
Total:	666							

**Table 2:** Risk factors for VIA-positive results among women from Tanzania, overall and according to HIV status.

	All Women (n = 672)				HIV-Positive (n = 103)				HIV-Negative (n = 564)			
	n	% VIA-Positive	OR *	95% CI	n	% VIA-Positive	OR †	95% CI	n	% VIA-Positive	OR ‡	95% CI
**Sociodemographic factors**												
Age												
18–24	46	8.70	1.011	0.281–3.640	4	50.00	4.477	0.386–51.918	42	4.76	0.719	0.147–3.524
25–34	193	15.54	2.308	1.106–4.818	20	40.00	2.477	0.606–10.124	173	12.72	1.902	0.830–4.355
35–44	223	12.11	1.634	0.793–3.366	38	31.58	1.606	0.461–5.599	184	8.15	1.223	0.512–2.923
≥45	165	8.48	1	-	31	16.13	1	-	132	6.82	1	-
Place of residence												
ORCI	353	11.9	1.338	0.665–2.690	42	40.48	3.847	0.997–14.840	310	8.06	1.053	0.440–2.519
Bagamoyo	161	14.29	1.651	0.771–3.500	25	24.00	2.263	0.498–10.283	134	12.69	1.744	0.692–4.392
Chalinze	120	9.17	1	-	29	13.79	1	-	91	7.69	1	-
Education level												
No formal education	62	16.13	2.360	0.567–9.826	11	27.27	0.926	0.088–9.768	51	13.73	2.545	0.620–10.457
Primary	366	10.11	1.207	0.357–4.074	59	22.03	0.635	0.088–4.604	305	7.87	1.367	0.396–4.716
Secondary	145	16.55	2.480	0.741–8.295	18	50.00	1.362	0.164–11.297	127	11.81	2.143	0.593–7.745
College/University	57	8.77	1	-	5	40.00	1	-	51	5.88	1	-
Household income												
Low	204	16.18	1.262	0.396–4.025	43	27.91	0.581	0.086–3.918	160	13.13	2.243	0.623–8.068
Medium	190	12.63	0.953	0.310–2.930	21	38.10	0.923	0.126–6.781	167	9.58	1.464	0.402–5.337
Variable	167	7.19	0.465	0.135–1.608	22	22.73	0.441	0.057–3.421	145	4.83	0.709	0.171–2.938
High	51	9.80	1	-	5	40.00	1	-	46	6.52	1	-
Religion												
Islam	325	12.92	1.165	0.720–1.887	50	26.00	0.728	0.297–1.786	274	10.58	1.397	0.769–2.538
Christianity	301	11.30	1	-	43	32.56	1	-	256	7.81	1	-
Alcohol												
Yes	103	13.59	1.144	0.609–2.148	15	33.33	1.250	0.375–4.170	88	10.23	1.090	0.506–2.350
Never	455	12.09	1	-	63	28.57	1	-	391	9.46	1	-
Not anymore	64	9.38	0.753	0.310–1.826	12	33.33	1.250	0.334–4.674	50	4.00	0.399	0.093–1.707
**Reproductive and sexual history**												
Pregnancy number												
≥6	92	11.96	1.326	0.672–2.620	10	20.00	0.563	0.080–3.939	81	11.11	1.347	0.509–3.565
4–5	154	10.39	0.945	0.436–2.051	19	26.32	0.804	0.169–3.819	135	8.15	0.956	0.381–2.399
2–3	243	13.99	1.107	0.472–2.600	48	33.33	1.125	0.300–4.219	193	9.33	1.109	0.480–2.562
0–1	119	10.92	1	-	13	30.77	1	-	106	8.49	1	-
First sex at age												
≤17	218	10.55	1.479	0.593–3.688	37	29.73	3.808	0.429–33.783	179	6.70	0.980	0.377–2.549
18–20	286	15.38	2.479	1.072–5.731	44	34.09	4.655	0.538–40.284	241	12.03	1.798	0.775–4.171
≥21	122	7.38	1	-	10	10.00	1	-	112	7.14	1	-
Sexual partner number												
≥5	98	16.33	1.717	0.806–3.658	29	41.38	2.118	0.196–22.900	69	5.80	0.567	0.179–1.792
2–4	373	11.80	1.177	0.633–2.187	57	24.56	0.977	0.094–10.162	313	9.58	0.977	0.501–1.904
1	147	10.20	1	-	4	25.00	1	-	143	9.79	1	-
Sex for money or gifts												
Yes	112	15.18	1.366	0.756–2.468	23	39.13	1.437	0.521–3.961	89	8.99	1.005	0.449–2.247
No	449	11.58	1	-	55	30.91	1	-	391	8.95	1	-
Birth control												
Yes	437	12.36	1.054	0.616–1.804	62	30.65	1.215	0.459–3.214	373	9.38	1.067	0.548–2.080
No	178	11.80	1	-	30	26.67	1	-	147	8.84	1	-
**Medical conditions**												
Diagnosed with STI												
Yes	56	16.07	1.382	0.647–2.951	19	36.84	1.342	0.460–3.912	37	5.41	0.533	0.124–2.291
No	534	12.17	1	-	66	30.30	1	-	465	9.68	1	-
Pap smear results												
HSIL	79	20.25	3.563	1.284–9.886	17	41.18	4.899	0.488–49.216	60	15.00	3.029	1.022–8.976
ASC-H	100	11.00	1.884	0.677–5.241	13	23.08	2.100	0.179–24.591	87	9.20	1.738	0.580–5.214
LSIL	170	14.12	2.485	0.999–6.178	28	32.14	3.315	0.353–31.151	142	10.56	2.028	0.760–5.412
ASCUS	163	10.43	1.655	0.630–4.350	30	23.33	2.130	0.222–20.405	132	7.58	1.407	0.495–4.003
NILM	117	5.98	1	-	8	12.50	1	-	109	5.50	1	-
HIV status												
HIV-positive	96	28.13	4.137	2.274–7.525	-	-	-	-	-	-	-	-
HIV-negative	535	9.16	1	-	-	-	-	-	-	-	-	-

* Adjusted for age, education level, household income, first sex age, PAP smear results and HIV status. † Adjusted for age, place of residence and education level. ‡ Adjusted for age, household income and first sex age.

**Table 3:** Performance of the VIA test in comparison to Pap smear among HIV+ and HIV− women at all sites. Significant values (calculated with Fischer’s Exact test) are shaded in gray.

	**Cohort (n = 421)**				**ORCI (n = 240)**			
	**HIV+**		**HIV−**		**HIV+**		**HIV−**	
	**n**	**%**	**n**	**%**	**n**	**%**	**n**	**%**
**Total**	63	14.96	358	85.04	27	11.25	213	88.75
		*p* < 0.0001				*p* < 0.0001		
**VIA+ HSIL** **−**	13	3.09	24	5.7	7	2.92	13	5.42
		*p* = 0.0011				*p* = 0.0029		
**VIA+ HSIL+**	6	1.42	7	1.66	3	1.25	2	0.83
		*p* = 0.0065				*p* = 0.0110		
**VIA** **−** **HSIL** **−**	37	8.79	281	66.75	16	6.67	173	72.08
		*p* = 0.0014				*p* = 0.0129		
**VIA** **−** **HSIL+**	7	1.67	46	10.93	1	0.42	25	10.42
		*p* = 0.8380				*p* = 0.3263		
	**Bagamoyo (n = 84)**				**Chalinze (n = 97)**			
	**HIV+**		**HIV** **−**		**HIV+**		**HIV** **−**	
	**n**	**%**	**n**	**%**	**n**	**%**	**n**	**%**
**Total**	15	17.86	69	82.14	21	21.65	76	78.35
		*p* = 0.0114				*p* = 0.1806		
**VIA+ HSIL** **−**	3	3.57	7	8.33	3	3.09	4	4.12
		*p* = 0.3741				*p* = 0.1704		
**VIA+ HSIL+**	3	3.57	2	2.38	0	0	3	3.09
		*p* = 0.0377				*p* = 1.0000		
**VIA** **−** **HSIL** **−**	6	7.14	54	64.29	11	11.34	54	55.67
		*p* = 0.0088				*p* = 0.1222		
**VIA** **−** **HSIL+**	3	3.57	6	7.14	7	7.22	15	15.46
		*p* = 0.1976				*p* = 0.2393		

## Data Availability

The data presented in this study is available upon reasonable request.
